# WNK Inhibition Increases Surface Liquid pH and Host Defense in Cystic Fibrosis Airway Epithelia

**DOI:** 10.1165/rcmb.2022-0172OC

**Published:** 2022-07-18

**Authors:** Tayyab Rehman, Philip H. Karp, Andrew L. Thurman, Steven E. Mather, Akansha Jain, Ashley L. Cooney, Patrick L. Sinn, Alejandro A. Pezzulo, Michael E. Duffey, Michael J. Welsh

**Affiliations:** ^1^ Department of Internal Medicine and; ^3^ Department of Pediatrics, Pappajohn Biomedical Institute,; ^5^ Department of Molecular Physiology and Biophysics, Roy J. and Lucille A. Carver College of Medicine, and; ^2^ Howard Hughes Medical Institute, University of Iowa, Iowa City, Iowa; and; ^4^ Department of Physiology and Biophysics, Jacobs School of Medicine and Biomedical Sciences, University at Buffalo, Buffalo, New York

**Keywords:** cystic fibrosis, WNK kinases, airway surface liquid, anion transport, pH

## Abstract

In cystic fibrosis (CF), reduced HCO_3_^−^ secretion acidifies the airway surface liquid (ASL), and the acidic pH disrupts host defenses. Thus, understanding the control of ASL pH (pH_ASL_) in CF may help identify novel targets and facilitate therapeutic development. In diverse epithelia, the WNK (with-no-lysine [K]) kinases coordinate HCO_3_^−^ and Cl^−^ transport, but their functions in airway epithelia are poorly understood. Here, we tested the hypothesis that WNK kinases regulate CF pH_ASL_. In primary cultures of differentiated human airway epithelia, inhibiting WNK kinases acutely increased both CF and non-CF pH_ASL_. This response was HCO_3_^−^ dependent and involved downstream SPAK/OSR1 (Ste20/SPS1-related proline-alanine-rich protein kinase/oxidative stress responsive 1 kinase). Importantly, WNK inhibition enhanced key host defenses otherwise impaired in CF. Human airway epithelia expressed two *WNK* isoforms in secretory cells and ionocytes, and knockdown of either *WNK1* or *WNK2* increased CF pH_ASL_. WNK inhibition decreased Cl^−^ secretion and the response to bumetanide, an NKCC1 (sodium-potassium-chloride cotransporter 1) inhibitor. Surprisingly, bumetanide alone or basolateral Cl^−^ substitution also alkalinized CF pH_ASL_. These data suggest that WNK kinases influence the balance between transepithelial Cl^−^ versus HCO_3_^−^ secretion. Moreover, reducing basolateral Cl^−^ entry may increase HCO_3_^−^ secretion and raise pH_ASL_, thereby improving CF host defenses.

Cystic fibrosis (CF) is an inherited, multisystem channelopathy caused by mutations in the *CFTR* (cystic fibrosis transmembrane conductance regulator) gene (1–3). Loss of CFTR protein function reduces anion secretion, disrupts epithelial function, and impairs airway host defense. These abnormalities result in chronic airway obstruction, inflammation, infection, tissue destruction, and bronchiectasis and limit the life span of affected individuals.

CFTR is an apical HCO_3_^−^ and Cl^−^ channel (4–6). In airway epithelia, these transport activities control the acid–base balance and composition of the thin film of liquid, the airway surface liquid (ASL), that covers the apical membrane. The ASL interfaces with the environment and mediates at least two vital respiratory host defenses (7–9). Mucociliary clearance uses gel-forming mucins to trap inhaled particles and ciliary beating to propel them out of the airways. Secreted antimicrobial peptides disrupt bacterial cell membranes and kill inhaled pathogens. An abnormally acidic pH of the ASL (pH_ASL_) resulting from reduced CFTR-mediated HCO_3_^−^ secretion impairs these respiratory defenses (10–18). Importantly, ASL alkalinization rescues these defects and may benefit individuals with CF independent of *CFTR* genotype (19–21).

Transepithelial HCO_3_^−^ secretion is a complex process. Several studies have identified key apical and basolateral transporters involved in this process (11, 22–25); others have resolved tissue-specific and species–specific differences (26–28). However, the cellular and molecular mechanisms that regulate airway HCO_3_^−^ secretion in humans remain incompletely defined. CF airways express apical HCO_3_^−^ channels and transporters other than CFTR (8, 29). Thus, identifying mechanisms that regulate non-CFTR HCO_3_^−^ secretion may suggest novel ways to increase CF pH_ASL_.

We considered that knowledge of HCO_3_^−^ transport in nonairway epithelia might yield insights relevant to CF airways. In several epithelia, the WNK (with-no-lysine [K]) kinases act as key regulators of anion transport (30, 31). WNK kinases are serine/threonine protein kinases that modify surface expression or activity of membrane transporters. In the pancreas, which shares similarities with airway HCO_3_^−^ transport, WNK kinases control ductal HCO_3_^−^ secretion (26, 32). In one study of mouse pancreatic duct, silencing of WNK kinases increased, and WNK expression decreased HCO_3_^−^ secretion (33). In other reports, these kinases were shown to modulate CFTR HCO_3_^−^ channel activity (34, 35) and membrane expression of SLC26 (solute carrier 26) family transporters (36). However, whether WNK kinases coordinate HCO_3_^−^ secretion across human airway epithelia remains poorly understood.

In this study, we tested the hypothesis that WNK kinases regulate CF pH_ASL_. We studied primary cultures of differentiated human airway epithelia and applied pharmacologic and genetic interventions to elicit responses. Our results show that airway epithelia express two WNK isoforms, WNK1 and WNK2, in secretory cells and ionocytes. Importantly, reducing WNK kinase activity increases pH_ASL_ and enhances key respiratory host defenses that are otherwise impaired in CF.

## Methods

Additional details on materials and methods are in the data supplement.

### Cell Culture

Airway epithelial cells were harvested from human lungs procured as postmortem specimens, as explants from patients undergoing lung transplant, or as lungs deemed unfit for transplant. Informed consent for use in research was obtained. All studies were approved by the University of Iowa Institutional Review Board. Proximal bronchi were dissected, cut into small pieces, and enzymatically digested. Epithelial cells were isolated and seeded without passage onto collagen-coated inserts (Costar, 3470; Falcon, 353180). Cell culture medium comprised a 1:1 mixture of Dulbecco’s modified Eagle medium/F-12, supplemented with 2% Ultroser G (Sartorius). Epithelia were differentiated at the air–liquid interface for 3 weeks or more before assay (37). During the course of this study, new CF lung donors became scarce, partly due to more individuals taking highly active CFTR modulators. To manage this situation, epithelial cells from previous donors with CF cryopreserved at P0 were thawed and differentiated. Table E1 in the data supplement reports genotypes of CF donors included in this study. Whenever feasible, studies followed a paired design so that epithelia from the same donor were assayed under control and treatment conditions. In experiments shown in Figure 2B, differentiated airway epithelia were generated from cryostocks of transformed human airway epithelial cell lines NuLi-1 (wild-type [WT]/WT) and CuFi-4 (G551D/ΔF508), as previously reported (38). These cell lines were used as additional models to test CFTR dependence of pH_ASL_ responses evoked by inhibiting WNK kinases. To assess cytokine-induced responses, epithelia were treated with a combination of 10 ng/ml TNFα (R&D Systems) and 20 ng/ml IL-17 (R&D Systems). Both cytokines were added to the Ultroser G-supplemented basolateral media for 48 hours before assessments.

### Pharmacologic Reagents

WNK463 and ivacaftor were purchased from Selleckchem. Other reagents were purchased from MilliporeSigma.

### Single-Cell RNA-seq and Analysis

Cells for scRNA-seq (single-cell RNA sequencing) were obtained from primary cultures of human airway epithelia. The epithelia were grown at the air–liquid interface for 3 weeks or more before assay. The cell culture methods were the same as reported above. At the time of assessment, all epithelia were visibly dry on the apical side. Electrophysiologic assessments in Ussing chambers showed a mean basal transepithelial conductance (G_t_) of 3.2 mS/cm^2^, resistance of 433.3 Ω.cm^2^, and short-circuit current (I_SC_) of 72.6 mA/cm^2^. These properties indicated well-differentiated, polarized epithelia performing electrogenic ion transport. Additional details about library preparation, sequencing methods, and bioinformatic analysis can be found in the supplement. The data are available in the National Center for Biotechnology Information’s Gene Expression Omnibus (GEO) database (GEO GSE159056).

### Immunocytochemistry

Airway epithelia were washed, fixed, permeabilized, and immunostained to reveal WNK1 and WNK2 expression. See supplemental methods for details.

### pH_ASL_ Measurement

pH_ASL_ was measured using a fluorescent ratiometric pH indicator, SNARF-1, conjugated to 70 kD dextran (Thermo Fisher Scientific). Additional details are reported in the supplement.

### Epithelial Host Defenses

Several assays were performed to assess epithelial defense mechanisms. *1*) ASL viscosity was measured using the fluorescence recovery after photobleaching method (12). *2*) Liquid absorption was measured using the micropipette technique (39). *3*) Ciliary beat frequency was measured using phase contrast microscopy. *4*) ASL antimicrobial activity was assessed using bacteria-coated grids (13). Additional details on these assays are provided in the supplement.

### siRNA Knockdown

Gene knockdown in primary CF airway epithelia was achieved as reported previously (40). siRNAs were obtained from Integrated DNA Technologies (negative control: IDT DS NC 1; *WNK1*: IDT hs.Ri.WNK1.13.2; *WNK2*: IDT hs.Ri.WNK2.13.3) and transfected into dissociated primary airway epithelial cells using Lipofectamine RNAiMax (Invitrogen). Transfected cells were seeded onto collagen-coated inserts (Costar, 3470) and differentiated at the air–liquid interface. pH_ASL_ was measured at Day 6 or 7 after seeding. The efficiency of gene knockdown was assessed with RT-PCR.

### Electrophysiologic Studies

Airway epithelia were mounted in modified Ussing chambers (Physiologic Instruments) and bathed in symmetric Krebs buffer solution. Epithelia were voltage clamped, followed by recording of the I_SC_ and G_t_. See supplemental information for details.

### Bulk RNA-seq

RNA isolation, library preparation, sequencing, and bioinformatics analysis were previously reported (41). RNA-seq data are available in the National Center for Biotechnology Information’s GEO database (GEO GSE176121).

### Real-Time PCR

The primer pairs used were as follows: *WNK1*, 5′-GCCGTCAGATCCTTAAAGGTC-3′ and 5′-CCAGTAGGGCCGGTGAT AA-3′; *WNK2*, 5′-CATACCTGAAGCG GTTCAAGG-3′ and 5′-CTTTTGGCAAATGACGCTCTTT-3′; and *SFRS9*, 5′-TGCGTAAACTGGATGACACC-3′ and 5′-CCTGCTTTGGTATGGAGAGTC-3′. See supplemental methods for details.

### Statistics

Statistical significance testing was performed on GraphPad Prism 8 Software. Statistical tests included paired Student’s *t* test for comparing two groups and one-way ANOVA with Tukey’s multiple comparison test for comparing more than two groups. A *P* value of <0.05 was considered significant.

## Results

### Airway Epithelia Express WNK1 and WNK2

The four *WNK* isoforms are expressed in a tissue-specific manner (42, 43). However, their expression in human airways remains relatively unexplored. Recent scRNA-seq studies have revealed considerable cellular-level heterogeneity within airway epithelia with implications for ion transport (44–46). Notably, these studies have shown that secretory cells express nearly half the epithelial *CFTR* transcript, and the ionocytes, though rare, express the highest amount on a per-cell basis (44–47). These cell types also express basolateral transporters involved in HCO_3_^−^ and Cl^−^ secretion.

**Figure 1.**
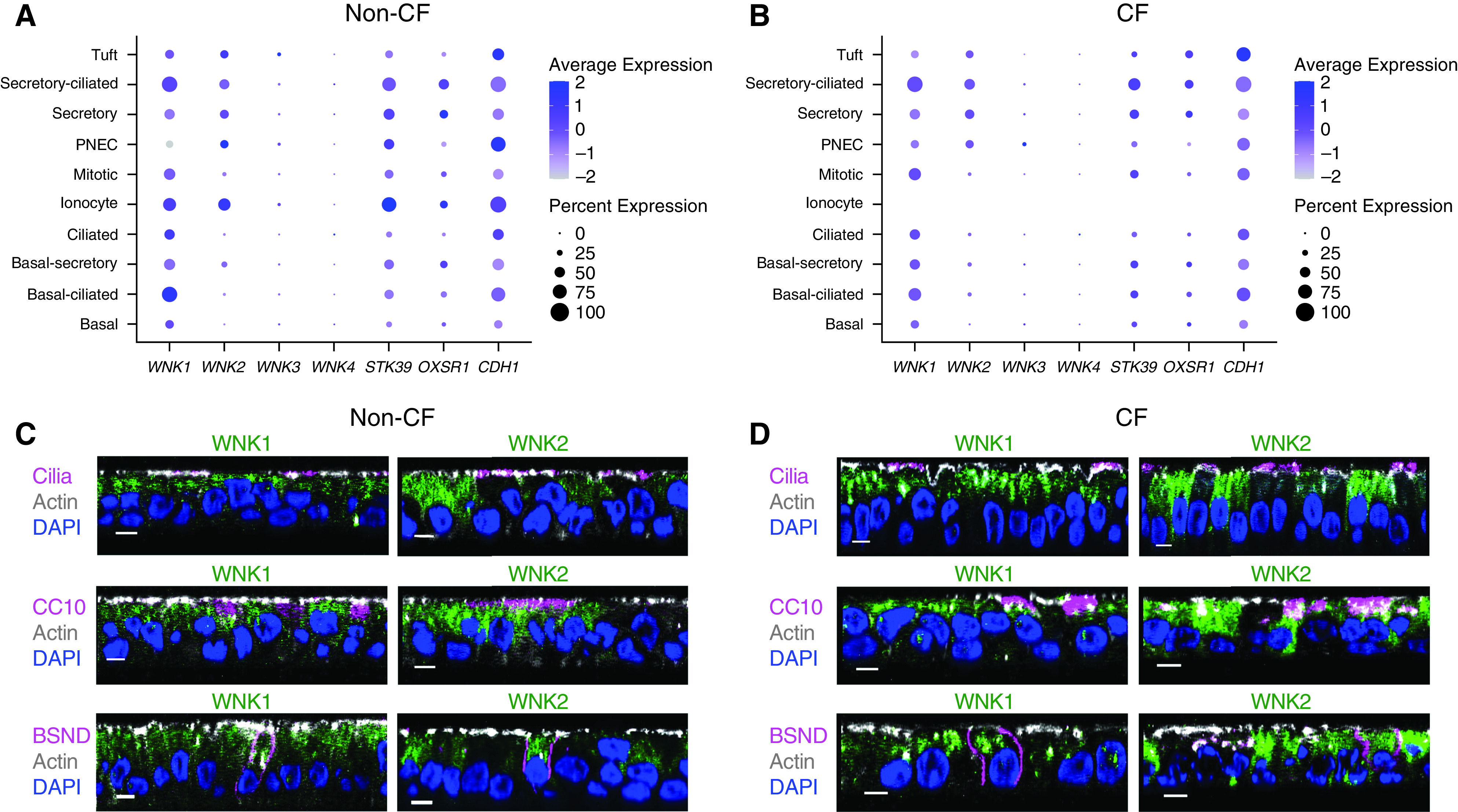
Expression of WNK (with-no-lysine [K]) kinases in human airway epithelia. (*A* and *B*) Single-cell RNA-seq was performed on primary cultures of differentiated airway epithelia from donors without (*n* = 4) and with cystic fibrosis (CF) [*n* = 4; *see* Table E1 for cystic fibrosis transmembrane conductance regulator (CFTR) genotypes]. Dot plot showing cell type–specific expression of the four *WNK* isoforms, and *STK39* (serine/threonine kinase 39) and *OXSR1* (oxidative stress responsive kinase 1), which encode Ste20/SPS1-related proline-alanine-rich protein kinase (SPAK) and oxidative stress responsive 1 kinase (OSR1), respectively. *CDH1* (e-cadherin) is included as a reference epithelial gene. For each dot, the size represents the detection rate in a particular cell type, and the color represents average gene expression for cells in which gene was detected. Data for CF ionocytes is not shown, as these cells were not detected in three out of four CF epithelia. Also see Figure E2. (*C* and *D*) Confocal images showing WNK1 and WNK2 immunolocalization in non-CF and CF epithelia. Scale bar, 5 μm. For each panel, similar staining results were obtained in two different donors. RNA-seq = RNA sequencing; PNEC = pulmonary neuroendocrine cell.

To identify which WNK kinases might regulate anion transport across airway epithelia, we performed scRNA-seq. We studied primary cultures of differentiated airway epithelia from four different donors without CF and four different donors with CF and examined cell type–specific *WNK* gene expression (Figures 1A and 1B). *WNK1* was broadly expressed in all major cell types (i.e., secretory cells, ciliated cells, and basal cells), as well as ionocytes. *WNK2* was also abundantly expressed in secretory cells and ionocytes. In contrast to *WNK1*, *WNK2* was rarely detected in ciliated or basal cells. The remaining *WNK* isoforms, *WNK3* and *WNK4*, were either not expressed, or expressed at a very low level. Importantly, *WNK* genes showed similar expression in CF versus non-CF epithelia (Figure E2). We also studied the expression of the two main downstream kinases (i.e., *STK39*, which encodes Ste20/SPS1-related proline-alanine-rich protein kinase [SPAK], and *OXSR1*, which encodes oxidative stress responsive 1 kinase [OSR1]). Both genes were broadly expressed and abundantly detected in secretory cells as well as ionocytes.

To reveal WNK protein expression, we immunolabeled non-CF and CF epithelia for WNK1 and WNK2 (Figures 1C and 1D). In agreement with scRNA-seq results, we detected WNK1 in ciliated as well as nonciliated cells and WNK2 predominantly in nonciliated cells. Further immunolocalization studies revealed WNK1 and WNK2 expression in secretory cells (labeled with anti-CC10 antibody) as well as ionocytes (labeled with anti-BSND antibody). Overall, these studies identified two WNK kinases in airway cells that secrete anions.

### WNK Inhibition Increases CF pH_ASL_

**Figure 2.**
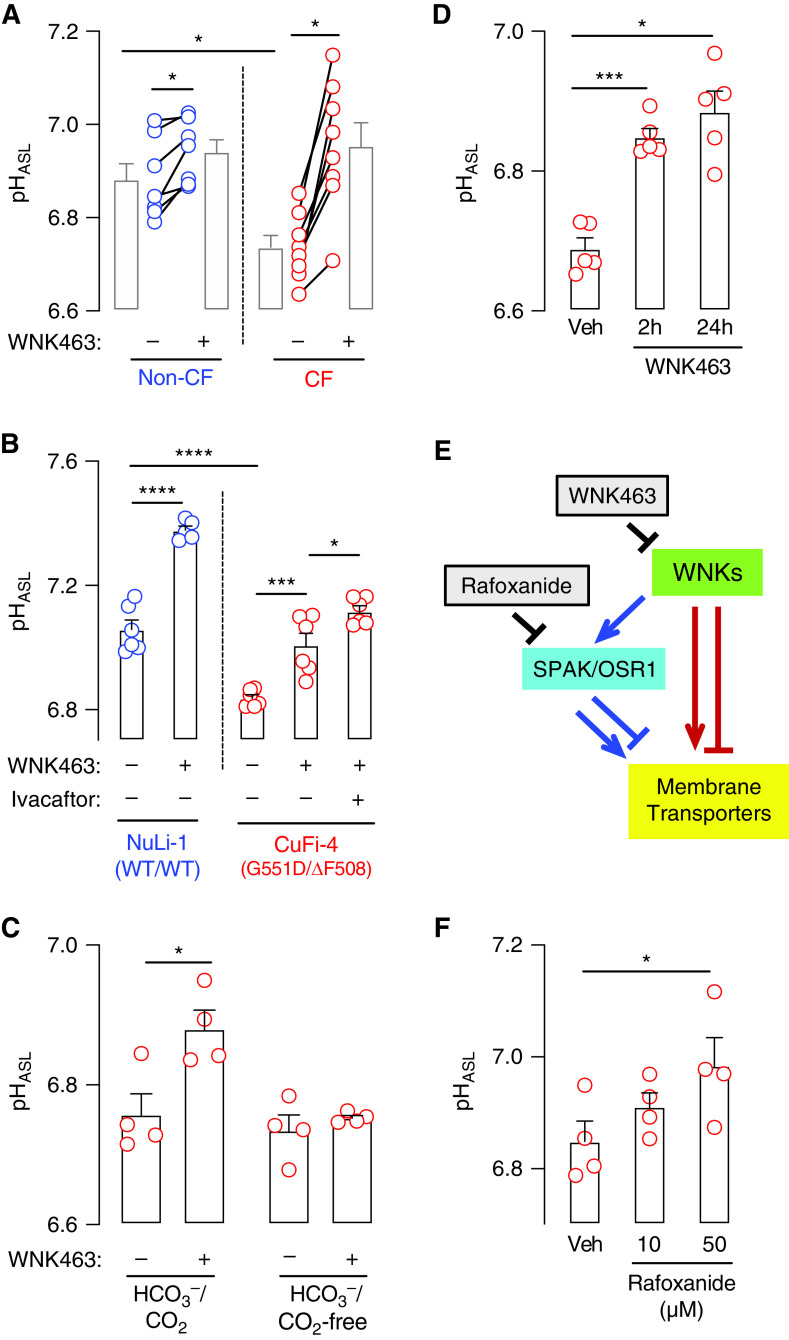
WNK463 increases airway surface liquid pH (pH_ASL_). Human airway epithelia were exposed to either vehicle or WNK463 (10 μM) for 2 hours, and pH_ASL_ was measured using SNARF-1-dextran. (*A*) pH_ASL_ responses in primary cultures of non-CF (*n* = 7) and CF epithelia (*n* = 8). (*B*) pH_ASL_ responses in NuLi-1 (*n* = 6) and CuFi-4 epithelia (*n* = 6). Ivacaftor (10 μM) was applied for 2 hours in combination with WNK463. (*C*) pH_ASL_ response in primary CF epithelia in the presence of HCO_3_^−^/CO_2_ and after replacing HCO_3_^−^ with HEPES and removing CO_2_ from the environment (*n* = 4). (*D*) Time course of WNK463-evoked response in primary CF epithelia (*n* = 5). (*E*) Schematic showing direct versus indirect modulation of membrane transporters by WNK kinases. (*F*) pH_ASL_ response in primary CF epithelia after 2-hour exposure to rafoxanide, a SPAK/OSR1 inhibitor (*n* = 4). In *B*, each data point is an epithelium derived from either NuLi-1 or CuFi-4 cells. In all other cases, each data point represents a primary differentiated airway epithelium from a different human donor. Data are shown as mean ± SEM. Statistical significance was tested using two-way ANOVA with two-stage Benjamini, Krieger, and Yekuteli false discovery rate procedure for *A*, and ANOVA with *post hoc* Tukey’s test for *B*, *C*, *D*, and *F*. *P < 0.05, ***P < 0.001, and ****P < 0.0001. Veh = vehicle.

Several HCO_3_^−^ and H^+^ transport mechanisms integrate to determine pH_ASL_, and pH_ASL_ influences host defense (13, 19). To begin to understand the role of WNK kinases in regulating pH_ASL_, we used pharmacologic WNK inhibition. WNK463 is a selective, ATP-competitive, pan-WNK kinase inhibitor and has recently emerged as a useful tool for studying ion transport physiology (48–51). We exposed airway epithelia to either vehicle or WNK463 for 2 hours and measured pH_ASL_ in an environment containing 25 mM HCO_3_^−^ and 5% CO_2_. In primary cultures of both CF and non-CF epithelia, WNK463 increased pH_ASL_ (Figure 2A). As an additional test, we also studied NuLi-1 (WT/WT) and CuFi-4 (G551D/ΔF508) epithelia and found that WNK463 elicited similar responses (Figure 2B). Alkalinization in CF epithelia indicated that the WNK463-induced response did not require CFTR. However, exposure of WNK463-treated CuFi-4 epithelia to ivacaftor further increased pH_ASL_. Ivacaftor increases open-state probability and function of CFTR-G551D channels. We concluded that inhibiting WNK supports ASL alkalinization through CFTR-independent as well as CFTR-dependent mechanisms.

To further characterize the WNK463-induced response, we performed additional studies in primary differentiated airway epithelia. When these epithelia were exposed to WNK463 in a nominally HCO_3_^−^/CO_2_-free environment, the pH_ASL_ response disappeared (Figure 2C). This result suggested that WNK463 increased CF pH_ASL_ by increasing HCO_3_^−^ secretion and not by decreasing H^+^ secretion. Next, we asked whether this response was time and dose dependent. Two hours of exposure increased pH_ASL_, and continued exposure up to 24 hours did not further alkalinize ASL (Figure 2D). Moreover, 10 μM WNK463 alkalinized but a lower dose (1 μM) did not alter pH_ASL_ (Figure E3).

WNK kinases modulate membrane transporters either directly or indirectly through their native substrates, SPAK and OSR1 (52) (Figure 2E). In scRNA-seq data, cell types expressing *WNK1* and *WNK2* also expressed genes encoding SPAK and OSR1. To test the latter’s involvement in controlling pH_ASL_, we treated CF epithelia with rafoxanide, an allosteric SPAK/OSR1 inhibitor (53). Similar to WNK463, rafoxanide applied for 2 hours also increased CF pH_ASL_ (Figure 2F). Taken together, these responses suggested that CF pH_ASL_ is controlled by upstream as well as downstream kinases in the canonical WNK/SPAK/OSR1 signaling pathway.

### WNK463 Enhances CF Host Defenses

**Figure 3.**
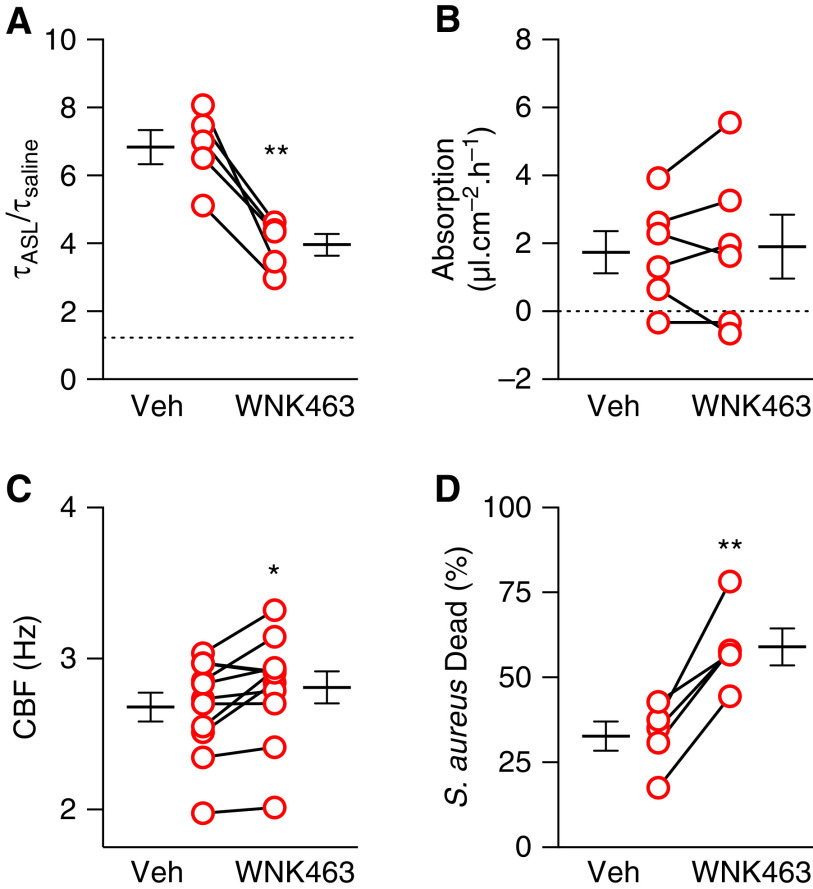
WNK463 enhances CF host defenses. Primary cultures of differentiated CF airway epithelia were treated with either vehicle or WNK463 (10 μM). All treatments were for 2 hours except *B*, where it was for 4 hours. (*A*) ASL viscosity (τ_ASL_/τ_saline_) in primary CF epithelia (*n* = 5). The dashed horizontal line indicates the viscosity of saline. (*B*) Rate of apical liquid absorption in primary CF epithelia (*n* = 6). The dashed horizontal line at 0 indicates no net secretion or absorption. (*C*) Ciliary beat frequency (CBF) in primary CF epithelia (*n* = 11). (*D*) ASL killing activity against *Staphylococcus aureus* in primary CF epithelia (*n* = 5). Each set of two data points with a connecting line represents epithelia from a different donor. Data are shown as mean ± SEM. Statistical significance was tested using paired Student’s *t* test. *P < 0.05 and **P < 0.01. Veh = vehicle.

Previous studies showed that alkalinizing CF ASL improves respiratory host defenses (12, 19–21). Because WNK463 increased pH_ASL_, we tested its impact in CF epithelia. Defective mucus transport is a key feature of CF (54). In primary cultures of differentiated CF epithelia, WNK463 decreased ASL viscosity (Figure 3A), consistent with previous studies showing that increasing pH_ASL_ decreases viscosity. In addition, WNK463 did not alter the rate of apical liquid absorption (Figure 3B), suggesting that a change in apical fluid volume was not involved. WNK463 also increased ciliary beat frequency, albeit modestly (Figure 3C). Both decrease in viscosity and increase in ciliary beat frequency would improve CF mucus transport. Previous studies also indicated that CF ASL has reduced antibacterial activity (13, 55). WNK463 increased ASL-mediated *Staphylococcus aureus* killing in primary CF epithelia (Figure 3D). Overall, these results suggested that targeting WNK kinase signaling may at least partially rescue CF host defense defects.

### Either *WNK1* or *WNK2* Knockdown Increases CF pH_ASL_

**Figure 4.**
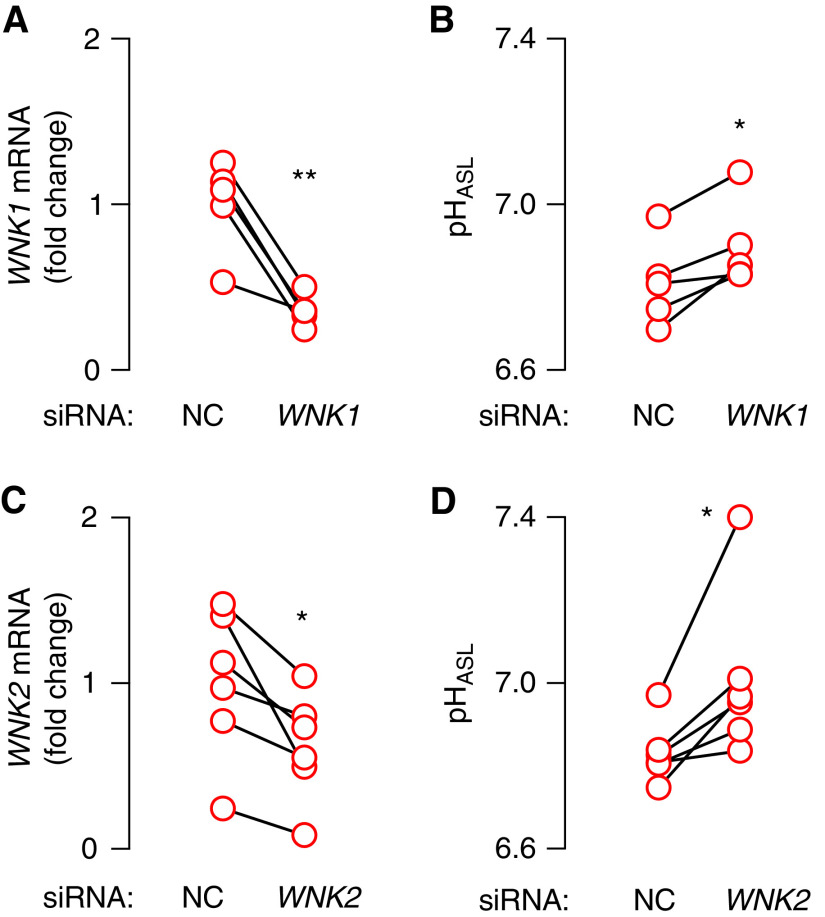
*WNK1* and *WNK2* regulate CF pH_ASL_. siRNAs were used to knock down gene expression in primary CF epithelia, and pH_ASL_ was measured using SNARF-1-dextran. Knockdown efficiency was assessed using qRT-PCR. (*A* and *B*) Knockdown of *WNK1* (*n* = 5). (*C* and *D*) Knockdown of *WNK2* (*n* = 6). Each data point represents an epithelium from a different donor. Statistical significance was tested using paired Student’s *t* test. *P < 0.05 and **P < 0.01. NC = negative control.

WNK463 is a pan-WNK kinase inhibitor (48). Because airway epithelia expressed two WNK kinases, we asked whether WNK1 or WNK2 controlled CF pH_ASL_. To test, we performed siRNA-mediated gene knockdown. Reducing either *WNK1* or *WNK2* expression increased CF pH_ASL_ (Figures 4A–4D). This result suggested that both isoforms, WNK1 and WNK2, participate in regulating CF pH_ASL_. Although analyses of additive and compensatory effects of WNK1 and WNK2 would require single- and double-knockout experiments rather than knockdown experiments, these knockdown studies suggest that if compensatory effects exist, they are incomplete.

### WNK463 Reduces Electrogenic Cl^−^ Secretion

**Figure 5.**
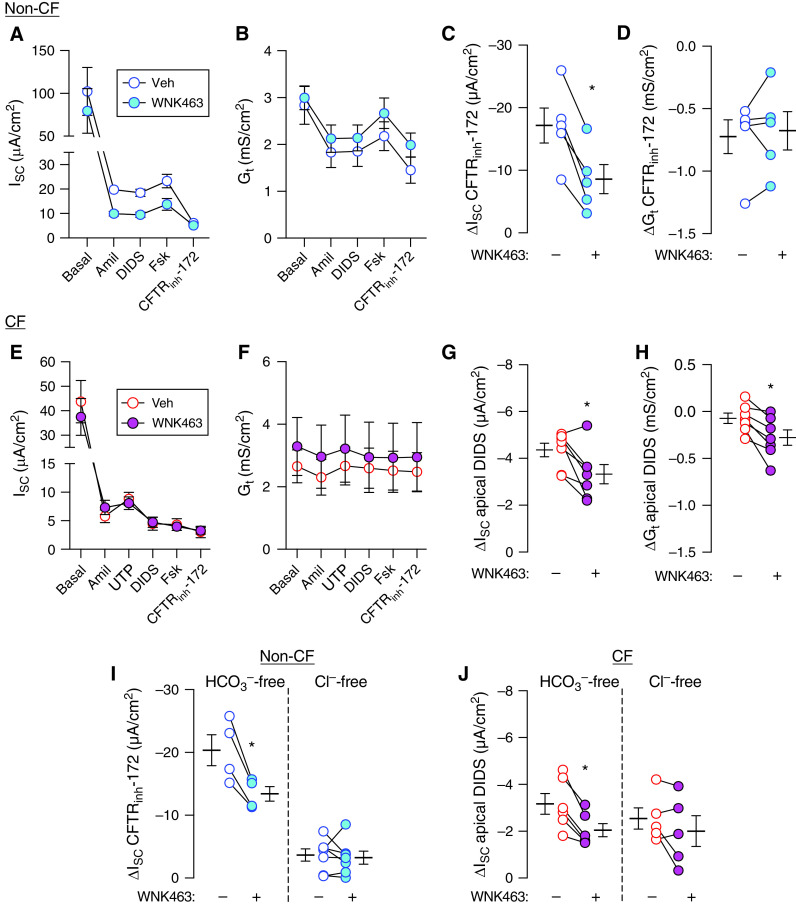
Effect of WNK463 on electrogenic Cl^−^ and HCO_3_^−^ secretion. Primary cultures of differentiated airway epithelia were treated with either vehicle (DMSO) or WNK463 (10 μM) for 2 hours. Epithelia were mounted in Ussing chambers and short-circuit current (I_SC_) and basal transepithelial conductance (G_t_) were recorded as agents were sequentially added to the apical side. (*A* and *B*) I_SC_ and G_t_ in non-CF epithelia (*n* = 5). (*C* and *D*) ΔI_SC_ and ΔG_t_ with addition of CFTR_inh_-172 in non-CF epithelia (*n* = 5). (*E* and *F*) I_SC_ and G_t_ in CF epithelia (*n* = 7). (*G* and *H*) ΔI_SC_ and ΔG_t_ with addition of apical DIDS (4,4′-diisothiocyano-2,2′-stilbenedisulfonic acid) in CF epithelia (*n* = 7). Studies in *A*–*H* were performed with symmetric buffers containing both Cl^−^ and HCO_3_^−^. (*I* and *J*) To separate the effect of WNK463 on electrogenic Cl^−^ versus HCO_3_^−^ transport, Ussing chamber studies were repeated with HCO_3_^−^-free or Cl^−^-free solutions. (*I*) shows ΔI_SC_ response with addition of CFTR_inh_-172 in non-CF epithelia (*n* = 4–7), and (*J*) shows ΔI_SC_ response with apical DIDS in CF epithelia (*n* = 5–6). Each data point represents an epithelium from a different donor. Data are shown as mean ± SEM. In some cases, error bars are hidden by symbols. Statistical significance was tested using paired Student’s *t* test. *P < 0.05. Veh = vehicle.

CFTR is the main route for anion exit across the apical membrane of airway epithelia. Whether WNK inhibition alters CFTR activity in airway epithelia is not well established. To test, we exposed non-CF epithelia to WNK463 for 2 hours and assayed in Ussing chambers containing symmetric Krebs solution (118 mM Cl^−^ and 25 mM HCO_3_^−^, gassed with 5% CO_2_). After clamping transepithelial voltage, we recorded I_SC_ and G_t_ and elicited responses to selective channel inhibitors or activators (Figures 5A and 5B). We added amiloride followed by DIDS (4,4′-diisothiocyano-2,2′-stilbenedisulfonic acid) to abolish ENaC (epithelial Na^+^ channel)-mediated Na^+^ absorption and CaCC (Ca^2+^-activated Cl^−^ channel)-mediated anion secretion, respectively. Next, we added forskolin to increase cellular cAMP and thereby phosphorylate and activate CFTR channels. We concluded with CFTR_inh_-172, an inhibitor of CFTR. We assessed the response to CFTR_inh_-172 and used it to estimate CFTR channel activity. WNK463 reduced ΔI_SC_-CFTR by ∼50% (Figure 5C). However, ΔG_t_-CFTR remained unchanged (Figure 5D). This result suggested that WNK463 decreased CFTR-mediated anion transport but did not alter CFTR channel activity at the apical membrane.

CF epithelia lack functional CFTR channels but express CaCC. Accordingly, we studied the effect of WNK463 on CaCC-mediated anion transport in CF epithelia (Figures 5E and 5F). After blocking ENaC with amiloride, we added uridine triphosphate, a P2Y2 purinergic receptor agonist that increases cytosolic [Ca^2+^] and thus activates CaCC. Next, we added DIDS, a nonspecific CaCC inhibitor, and recorded the change in I_SC_ and G_t_. WNK463 decreased DIDS-sensitive ΔI_SC_ but slightly increased DIDS-sensitive ΔG_t_ (Figures 5G and 5H). Together, these findings suggested that inhibiting WNK kinases reduces anion secretion, but the effect is not on apical anion channels.

To separate the effects of WNK inhibition on Cl^−^ versus HCO_3_^−^ transport, we repeated the studies in single anion solutions. In symmetric HCO_3_^−^-free solution, WNK463 reduced ΔI_SC_-CFTR (Figure 5I); however, in Cl^−^-free solution, ΔI_SC_-CFTR remained unchanged. Similar results were obtained for the DIDS-sensitive ΔI_SC_ in CF epithelia (Figure 5J). These data suggested that WNK inhibition reduces electrogenic Cl^−^ secretion but does not alter electrogenic HCO_3_^−^ secretion.

### Reducing Basolateral Cl^−^ Entry Increases CF pH_ASL_

**Figure 6.**
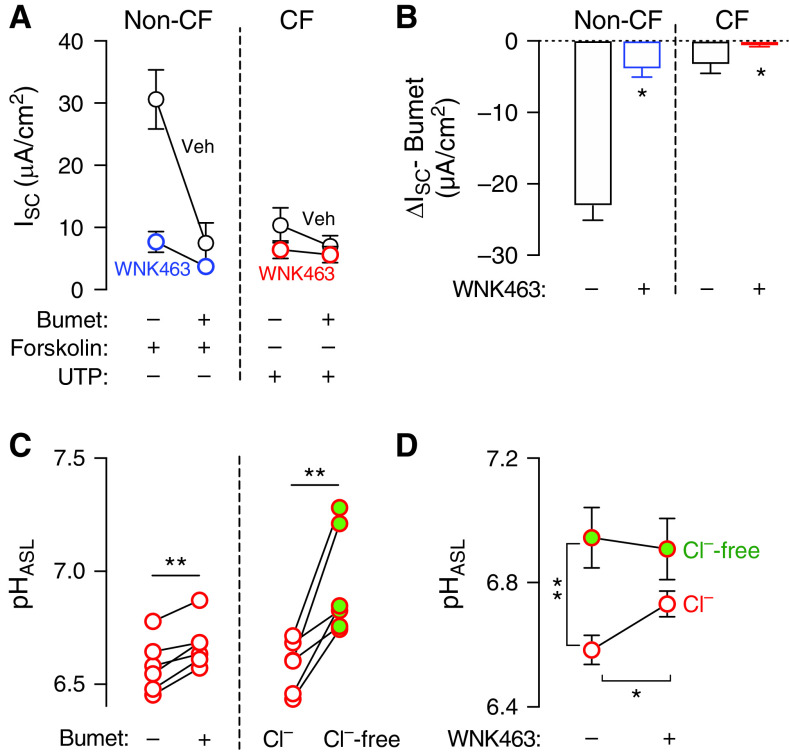
Reducing Cl^−^ transport increases CF pH_ASL_. (*A* and *B*) Primary cultures of differentiated airway epithelia were treated with either vehicle or WNK463 (10 μM) for 2 hours and assayed in Ussing chambers filled with symmetric Krebs buffer containing both Cl^−^ and HCO_3_^−^. During I_SC_ recording, amiloride was added to inhibit ENaC (epithelial Na^+^ channel), followed by forskolin (non-CF) or UTP (uridine triphosphate) (CF) to maximally activate CFTR or CaCC (Ca^2+^-activated Cl^−^ channel), respectively. At this point, basolateral bumetanide was introduced, and ΔI_SC_ was recorded (*n* = 6 different non-CF, or 9 different CF donors). (*C*) Left panel: CF pH_ASL_ response 2 hours after exposure to basolateral bumetanide (*n* = 6 different donors); right panel: CF pH_ASL_ in the presence or absence of basolateral Cl^−^ (*n* = 6 different donors). (*D*) CF pH_ASL_ response in epithelia exposed to WNK463 (10 μM) for 2 hours in the presence or absence of basolateral Cl^−^ (*n* = 6 different donors). Data are shown as mean ± SEM. Statistical significance was tested using paired Student’s *t* test. *P < 0.05 and **P < 0.01. Burnett = basolateral bumetanide.

Transcellular Cl^−^ secretion involves the movement of Cl^−^ across the apical and the basolateral membranes in series. Because studies of electrically conductive anion transport showed reduced Cl^−^ secretion without major effects at the apical membrane, we asked whether a change at the basolateral membrane was involved. The loop-sensitive NKCC (Na^+^-K^+^-2 Cl^−^) cotransporter is the main route for Cl^−^ entry across the basolateral membrane, and WNK kinases are known to increase NKCC activity in renal epithelia (56, 57). To further investigate the effect of WNK463 on this transport mechanism, we studied non-CF and CF epithelia in Ussing chambers. After blocking ENaC with amiloride, we added either forskolin to activate CFTR in non-CF epithelia, or uridine triphosphate to activate CaCC in CF epithelia. To estimate the contribution of NKCC1(sodium-potassium-chloride cotransporter 1), we added basolateral bumetanide and measured ΔI_SC_. WNK463 reduced bumetanide-sensitive I_SC_ in both non-CF and CF epithelia (Figures 6A and 6B). This result pointed to the involvement of WNK kinases in controlling basolateral Cl^−^ uptake through a bumetanide-sensitive mechanism.

Previous studies in airway epithelia showed that bumetanide decreases intracellular [Cl^−^] (58–60). This led us to hypothesize that lowering intracellular [Cl^−^] might also increase CF pH_ASL_. To test, we performed two experiments: *1*) We tested the effect of NKCC1 inhibition in CF epithelia. Exposure to bumetanide increased CF pH_ASL_ (Figure 6C, left panel). *2*) We measured pH_ASL_ in a Cl^−^-free environment. Similar to bumetanide, the removal of basolateral Cl^−^ also increased CF pH_ASL_ (Figure 6C, right panel). Because WNK463 reduced bumetanide-sensitive I_SC_, and lowering NKCC1 activity or intracellular [Cl^−^] increased pH_ASL_, we considered whether intracellular [Cl^−^] was involved in the response evoked by WNK463. When introduced in the absence of Cl^−^, WNK463 failed to alkalinize CF ASL, thus indicating that the WNK463-elicited pH_ASL_ response was Cl^−^ dependent (Figure 6D).

### WNK463 Further Increases pH_ASL_ in TNFα/IL-17–treated CF Epithelia

**Figure 7.**
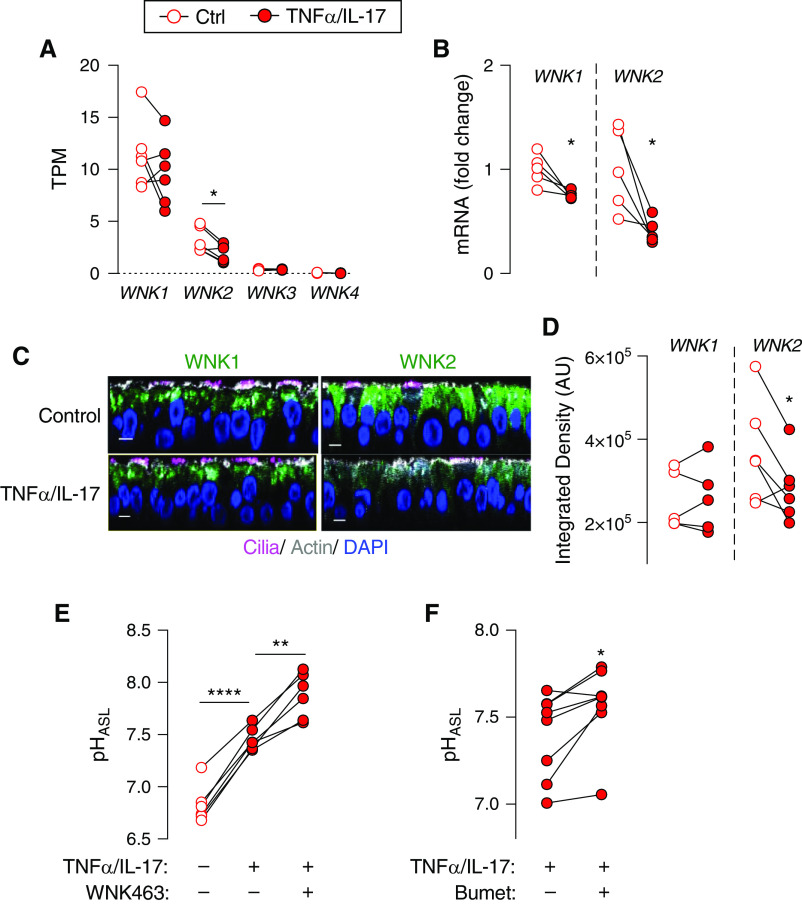
pH_ASL_ response to WNK463 in TNFα/IL-17–treated CF epithelia. Primary cultures of differentiated CF epithelia were treated with TNFα (10 ng/ml) and IL-17 (20 ng/ml) for 48 hours. (*A*) Changes in *WNK* gene expression revealed by bulk RNA-seq (*n* = 6). TPM = transcripts per million. (*B*) *WNK1* and *WNK2* mRNA expression measured using qRT-PCR (*n* = 5). (*C*) Immunostaining for WNK1 and WNK2 in control and TNFα/IL-17–treated CF epithelia. Scale bar, 5 μm. (*D*) Intensity of WNK1 or WNK2 immunolabeling quantitated as integrated density using imageJ software (*n* = 5–6). (*E* and *F*) pH_ASL_ responses in TNFα/IL-17–treated CF epithelia. WNK463 (10 μM) or bumetanide (100 μM) were applied for 2 hours before pH_ASL_ measurement (*n* = 6–8). Each data point represents an epithelium from a different donor. Data are shown as mean ± SEM. Statistical significance was tested using paired Student’s *t* test (*A*, *B*, *D*, and *F*) or ANOVA with *post* *hoc* Tukey’s test (*E*). *P < 0.05, **P < 0.01, and ****P < 0.0001.

Airway inflammation is ubiquitous in individuals with CF after the first few weeks of life (61–63). The CF airway inflammation is characteristically neutrophil predominant, may develop in the absence of infection, and is further exacerbated by infection and colonization. Two CF-relevant inflammatory cytokines, TNFα and IL-17, drive neutrophilic inflammation (64–67). In previous work, combined TNFα/IL-17 increased HCO_3_^−^ secretion and CF pH_ASL_ by increasing pendrin expression (41, 68). We asked if TNFα/IL-17–induced alkalinization was also accompanied by altered expression of WNK kinases. In gene expression studies, TNFα/IL-17 modestly reduced *WNK1* and markedly reduced *WNK2* expression (Figures 7A and 7B). In immunocytochemistry studies, TNFα/IL-17 decreased WNK2 detection, but WNK1 remained unchanged (Figures 7C and 7D). This led us to hypothesize that residual WNK kinases might continue to regulate HCO_3_^−^ secretion in cytokine-treated epithelia. Accordingly, exposure to WNK463 further increased pH_ASL_ in CF epithelia treated with TNFα/IL-17 (Figure 7E). Because WNK463 decreased Cl^−^ secretion, and reducing basolateral Cl^−^ entry increased CF pH_ASL_, we predicted a similar response to lowering basolateral Cl^−^ entry in cytokine-treated CF epithelia. Exposure to bumetanide further alkalinized ASL in TNFα/IL-17–treated CF epithelia (Figure 7F). This result suggested that TNFα/IL-17 shifted apical anion secretion in favor of HCO_3_^−^ over Cl^−^, and lowering basolateral Cl^−^ entry further augmented this response.

## Discussion

**Figure 8.**
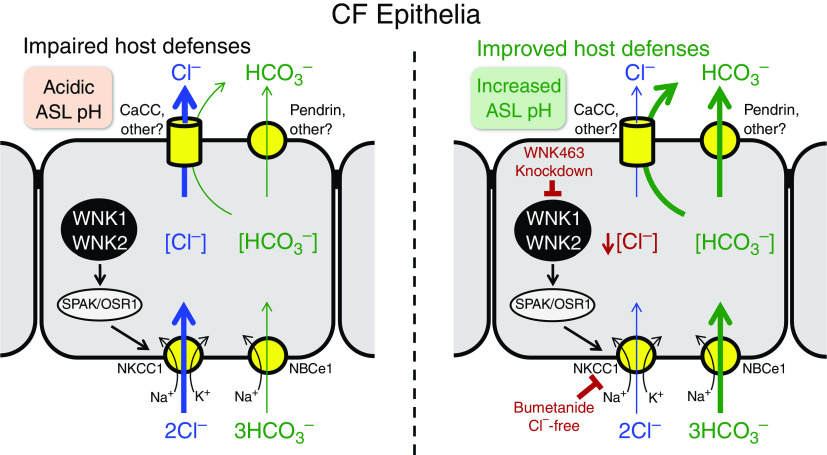
WNK kinases may regulate the ratio of HCO_3_^−^ versus Cl^−^ secretion in CF airway epithelia. Model shows a CF airway epithelial cell in which the absence of CFTR function decreases HCO_3_^−^ and Cl^−^ secretion, generates an acidic pH_ASL_, and impairs respiratory host defenses. Left panel: WNK1 and WNK2 regulate activity of basolateral NKCC1 (sodium-potassium-chloride cotransporter 1) via intermediate SPAK/OSR1 (Ste20/SPS1-related proline-alanine-rich protein kinase/oxidative stress responsive 1 kinase). NKCC1 imports Cl^−^ into the cell. Cl^−^ and HCO_3_^−^ exit from the cell across the apical membrane via channels and/or transporters whose identity and quantitative contributions are uncertain (indicated by question marks). Right panel: reducing WNK kinase activity, inhibiting NKCC1, and eliminating Cl^−^ lower the intracellular chloride concentration and transepithelial Cl^−^ secretion. At the same time, these interventions increase HCO_3_^−^ secretion, raise pH_ASL_, and improve CF host defenses. We speculate that intracellular Cl^−^ acts as a signaling molecule that regulates apical and/or basolateral HCO_3_^−^ transporters. Some features of this model are tentative at present and are opportunities for future research. See text for details.

Our transcript and immunocytochemistry data for *WNK1* and *WNK2*, and their substrates *STK39* and *OXSR1*, indicated that these kinases are expressed in secretory cells and ionocytes, the main airway epithelial cells that secrete anions. Consistent with that localization, pharmacologically inhibiting WNK kinases, SPAK/OSR1 kinases, and knocking down *WNK1* and *WNK2* transcripts increased pH_ASL_. These results thus identified an important role for WNK kinases in regulating HCO_3_^−^ secretion across airway epithelia. Figure 8 shows a tentative model for how WNK1 and WNK2 may influence Cl^−^ and HCO_3_^−^ secretion and the ratio between the two transport processes in CF airway epithelia. Some features in this model are unknown at present and are an opportunity for future research.

Electrophysiological studies indicated that inhibiting WNK kinases decreased the Cl^−^-mediated, but not HCO_3_^−^-mediated current. Moreover, ASL alkalinization persisted in the absence of CFTR activity in CF epithelia. A clue to a potential mechanism came with the finding that inhibiting WNK kinases largely eliminated the inhibitory effect of basolateral bumetanide on I_SC_. Bumetanide inhibits NKCC1, the major pathway for Cl^−^ entry into the cell, and thereby reduces the intracellular [Cl^−^] (58–60). Further evidence implicating intracellular [Cl^−^] came from studies showing that adding bumetanide alone or removing Cl^−^ from the medium also alkalinized ASL in CF epithelia.

These results suggest that WNK kinases may play a key role in determining the balance between Cl^−^ secretion and HCO_3_^−^ secretion across airway epithelia (Figure 8). Inhibiting WNK kinases decreased NKCC1 activity, which decreased Cl^−^ secretion, increased HCO_3_^−^ secretion, and increased pH_ASL_. The inference that inhibiting WNK reduces NKCC1 activity is supported by the finding that bumetanide also increased pH_ASL_ and previous reports that WNK kinases increase NKCC activity in nonairway epithelia (56, 57). However, the mechanism that increases HCO_3_^−^ secretion is uncertain. One possibility is that WNK inhibition reduces the intracellular [Cl^−^], thereby increasing the driving force for Cl^−^/ HCO_3_^−^ exchange at the apical membrane and hence HCO_3_^−^ secretion. Finding that bumetanide replicates the effect of WNK inhibition on pH_ASL_ is consistent with this hypothesis. However, Cl^−^-free bathing solution also induced HCO_3_^−^ secretion despite the fact that reduced [Cl^−^] would initially and transiently drive Cl^−^/HCO_3_^−^ exchange in the opposite direction, and over the 2-hour time course of the experiment, cellular Cl^−^ would be largely depleted. Thus, we favor an alternative explanation that intracellular Cl^−^ is a signaling molecule that regulates membrane transport (69).

Intracellular [Cl^−^] regulation of HCO_3_^−^ secretion has been reported previously. A Cl^−^-sensing motif has been identified in some HCO_3_^−^ transporters and other proteins (35, 70). Low intracellular [Cl^−^] was shown to increase IRBIT (IP3 receptor binding protein released with IP3)-stimulated NBCe1-B (electrogenic sodium bicarbonate cotransporter 1) activity (70). Kim and colleagues showed that low intracellular [Cl^−^] enabled structural association between WNK1 and CFTR and increased CFTR HCO_3_^−^ channel activity (35). Notably, this effect did not depend on WNK1 kinase activity. Yamaguchi and colleagues developed a computational model of guinea pig pancreatic duct HCO_3_^−^ secretion (71). In this model, maximal HCO_3_^−^ secretion did not depend on an increase in CFTR HCO_3_^−^ permeability or a change in SLC26 Cl^−^/HCO_3_^−^ exchange stoichiometry, but instead depended on suppression of basolateral Cl^−^ uptake. The addition of NKCC1, normally missing from guinea pig pancreatic ducts, increased intracellular [Cl^−^] and reduced secreted [HCO_3_^−^]. Our results also support intracellular [Cl^−^]-dependent regulation of HCO_3_^−^ secretion. It will be important for future studies to establish underlying molecular mechanisms in airway epithelia.

We previously reported that combined TNFα/IL-17 increased production of pendrin, an apical Cl^−^/ HCO_3_^−^ exchanger, and alkalinized CF ASL (41, 68). Here, we show that TNFα/IL-17 also reduced WNK2 expression. Moreover, inhibiting the residual WNK kinase activity with WNK463 further increased pH_ASL_, and basolateral bumetanide mimicked the effect of WNK inhibition. These proinflammatory cytokines may thus induce HCO_3_^−^ secretion and increase pH_ASL_ by at least two mechanisms, increasing pendrin expression and reducing WNK2 expression. Whether WNK kinases regulate apical expression or activity of pendrin in cytokine-treated airway epithelia remains to be determined. Some reports have suggested an interaction between pendrin and apical anion channels that may increase activity of both transporters (72); additional studies are needed to fully understand whether WNK kinases modulate such interactions.

WNK kinases have an ATP-binding site that is unique among protein kinases, and WNK463 targets this site. Previous studies have found WNK463 to be highly selective (48), but it is possible that higher doses may also affect other targets. To confirm the effect of reducing WNK activity on pH_ASL_, we used two orthogonal approaches (i.e., pharmacologic inhibition and gene knockdown). Moreover, inhibiting downstream WNK targets (i.e., SPAK/OSR1 and NKCC1) also increased pH_ASL_. Overall, these results point to a key role for the WNK signaling pathway in controlling airway HCO_3_^−^ secretion and pH_ASL_.

This study has several advantages. First, we studied primary cultures of differentiated human airway epithelia from both CF and non-CF genotypes. Second, to account for biological variability, we included epithelia from multiple human donors. Third, we measured pH_ASL_ under thin-film conditions without adding additional apical fluid. Fourth, in testing our hypothesis, we used a combination of pharmacologic, transcriptomic, gene silencing, protein immunolabeling, and electrophysiologic approaches. Fifth, although all studies and interventions were performed in primary cultures of differentiated airway epithelia, a preliminary study in established human airway epithelial cell lines, NuLi-1 and CuFi-4, yielded similar results. Finding that WNK signaling is active in these epithelia enables their use as models for studying WNK signaling.

This study also has limitations. First, we used human airway epithelia as an *in vitro* model, and assessing WNK kinase inhibition *in vivo* may be of value. However, interpretation of *in vivo* effects may be complicated by the fact that WNK kinases are expressed broadly in epithelial and nonepithelial cells (42, 43). Newer animal models with tissue- or cell-specific WNK knockouts might help further elucidate roles of WNK kinases. Second, we did not identify the transporter directly responsible for apical HCO_3_^−^ exit. RNA-seq studies show that CF airway epithelia express several non-CFTR HCO_3_^−^ transporters, including CaCC and SLC26 family members (41, 73), and WNK inhibition may affect more than one simultaneously. Third, inflammation in CF airways is a complex process, and it will be important for future studies to characterize the effects of other proinflammatory mediators (e.g., IL-8, IL-1β, etc.) on WNK signaling.

The results have implications for CF airways. First, previous studies have shown that the abnormally acidic pH_ASL_ observed in newborns with CF increases with time and inflammation (74, 75), although not to levels observed in non-CF epithelia studied under comparable conditions (41). As indicated above, our current data, together with previous results, suggest complex regulatory mechanisms are responsible. Second, loop diuretics, which inhibit NKCC, are commonly used to treat heart failure and fluid overload states (76). Yet, to our knowledge, these agents have not been shown to cause adverse airway phenotypes. This study suggests that HCO_3_^−^ secretion may compensate for any decrease in loop-sensitive Cl^−^ secretion and preserve, if not augment, host defenses (77). Third, by enhancing respiratory host defense, WNK inhibition might be a potential therapeutic target in CF and possibly in acquired CFTR dysfunction, such as that induced by cigarette smoking (78). Although WNK463 produced adverse effects in a rat model of hypertension (48), it is a nonselective WNK kinase inhibitor. Interestingly, WNK2 has a more restricted tissue expression than the ubiquitous WNK1, it is detected in airway epithelial cell types relevant for anion secretion, and its knockdown alkalinizes CF ASL. Thus, selective WNK2 inhibitors, inhibitors of downstream SPAK/OSR1 kinases, or inhibitors restricted to the airways might be pursued as potential CF therapeutics.

## References

[1] Quinton PM Physiological basis of cystic fibrosis: a historical perspective *Physiol Rev* 1999 79 S3 S22 9922374 10.1152/physrev.1999.79.1.S3

[2] Ratjen F Bell SC Rowe SM Goss CH Quittner AL Bush A Cystic fibrosis *Nat Rev Dis Primers* 2015 1 15010 27189798 10.1038/nrdp.2015.10 PMC7041544

[3] Stoltz DA Meyerholz DK Welsh MJ Origins of cystic fibrosis lung disease *N Engl J Med* 2015 372 1574 1575 10.1056/NEJMc1502191 25875271

[4] Poulsen JH Fischer H Illek B Machen TE Bicarbonate conductance and pH regulatory capability of cystic fibrosis transmembrane conductance regulator *Proc Natl Acad Sci USA* 1994 91 5340 5344 7515498 10.1073/pnas.91.12.5340 PMC43990

[5] Tang L Fatehi M Linsdell P Mechanism of direct bicarbonate transport by the CFTR anion channel *J Cyst Fibros* 2009 8 115 121 19019741 10.1016/j.jcf.2008.10.004

[6] Sheppard DN Welsh MJ Structure and function of the CFTR chloride channel *Physiol Rev* 1999 79 S23 S45 9922375 10.1152/physrev.1999.79.1.S23

[7] Widdicombe JH Airway epithelium Colloquium series on integrated systems physiology #36 San Rafael, CA Morgan & Claypool Life Sciences 2013

[8] Haq IJ Gray MA Garnett JP Ward C Brodlie M Airway surface liquid homeostasis in cystic fibrosis: pathophysiology and therapeutic targets *Thorax* 2016 71 284 287 26719229 10.1136/thoraxjnl-2015-207588

[9] Verkman AS Song Y Thiagarajah JR Role of airway surface liquid and submucosal glands in cystic fibrosis lung disease *Am J Physiol Cell Physiol* 2003 284 C2 C15 12475759 10.1152/ajpcell.00417.2002

[10] Gustafsson JK Ermund A Ambort D Johansson ME Nilsson HE Thorell K *et al.* Bicarbonate and functional CFTR channel are required for proper mucin secretion and link cystic fibrosis with its mucus phenotype *J Exp Med* 2012 209 1263 1272 22711878 10.1084/jem.20120562 PMC3405509

[11] Zajac M Dreano E Edwards A Planelles G Sermet-Gaudelus I Airway surface liquid pH regulation in airway epithelium current understandings and gaps in knowledge *Int J Mol Sci* 2021 22 3384 33806154 10.3390/ijms22073384 PMC8037888

[12] Tang XX Ostedgaard LS Hoegger MJ Moninger TO Karp PH McMenimen JD *et al.* Acidic pH increases airway surface liquid viscosity in cystic fibrosis *J Clin Invest* 2016 126 879 891 26808501 10.1172/JCI83922 PMC4767348

[13] Pezzulo AA Tang XX Hoegger MJ Abou Alaiwa MH Ramachandran S Moninger TO *et al.* Reduced airway surface pH impairs bacterial killing in the porcine cystic fibrosis lung *Nature* 2012 487 109 113 22763554 10.1038/nature11130 PMC3390761

[14] Abou Alaiwa MH Reznikov LR Gansemer ND Sheets KA Horswill AR Stoltz DA *et al.* pH modulates the activity and synergism of the airway surface liquid antimicrobials β-defensin-3 and LL-37 *Proc Natl Acad Sci USA* 2014 111 18703 18708 25512526 10.1073/pnas.1422091112 PMC4284593

[15] Simonin J Bille E Crambert G Noel S Dreano E Edwards A *et al.* Airway surface liquid acidification initiates host defense abnormalities in cystic fibrosis *Sci Rep* 2019 9 6516 31019198 10.1038/s41598-019-42751-4 PMC6482305

[16] Quinton PM Cystic fibrosis: impaired bicarbonate secretion and mucoviscidosis *Lancet* 2008 372 415 417 18675692 10.1016/S0140-6736(08)61162-9

[17] Nakayama K Jia YX Hirai H Shinkawa M Yamaya M Sekizawa K *et al.* Acid stimulation reduces bactericidal activity of surface liquid in cultured human airway epithelial cells *Am J Respir Cell Mol Biol* 2002 26 105 113 11751210 10.1165/ajrcmb.26.1.4425

[18] Birket SE Davis JM Fernandez CM Tuggle KL Oden AM Chu KK *et al.* Development of an airway mucus defect in the cystic fibrosis rat *JCI Insight* 2018 3 97199 29321377 10.1172/jci.insight.97199 PMC5821204

[19] Shah VS Meyerholz DK Tang XX Reznikov L Abou Alaiwa M Ernst SE *et al.* Airway acidification initiates host defense abnormalities in cystic fibrosis mice *Science* 2016 351 503 507 26823428 10.1126/science.aad5589 PMC4852973

[20] Abou Alaiwa MH Launspach JL Sheets KA Rivera JA Gansemer ND Taft PJ *et al.* Repurposing tromethamine as inhaled therapy to treat CF airway disease *JCI Insight* 2016 1 87535 27390778 10.1172/jci.insight.87535 PMC4933331

[21] Muraglia KA Chorghade RS Kim BR Tang XX Shah VS Grillo AS *et al.* Small-molecule ion channels increase host defences in cystic fibrosis airway epithelia *Nature* 2019 567 405 408 30867598 10.1038/s41586-019-1018-5 PMC6492938

[22] Sinđić A Sussman CR Romero MF Primers on molecular pathways: bicarbonate transport by the pancreas *Pancreatology* 2010 10 660 663 21242704 10.1159/000323435 PMC3068561

[23] Fischer H Widdicombe JH Mechanisms of acid and base secretion by the airway epithelium *J Membr Biol* 2006 211 139 150 17091214 10.1007/s00232-006-0861-0 PMC2929530

[24] Cordat E Casey JR Bicarbonate transport in cell physiology and disease *Biochem J* 2009 417 423 439 19099540 10.1042/BJ20081634

[25] Alka K Casey JR Bicarbonate transport in health and disease *IUBMB Life* 2014 66 596 615 25270914 10.1002/iub.1315

[26] Lee MG Ohana E Park HW Yang D Muallem S Molecular mechanism of pancreatic and salivary gland fluid and HCO3 secretion *Physiol Rev* 2012 92 39 74 22298651 10.1152/physrev.00011.2011 PMC3667394

[27] Shah VS Chivukula RR Lin B Waghray A Rajagopal J Cystic fibrosis and the cells of the airway epithelium: what are ionocytes and what do they do? *Annu Rev Pathol* 2022 17 23 46 34437820 10.1146/annurev-pathol-042420-094031 PMC10837786

[28] Kunzelmann K Schreiber R Hadorn HB Bicarbonate in cystic fibrosis *J Cyst Fibros* 2017 16 653 662 28732801 10.1016/j.jcf.2017.06.005

[29] Li H Salomon JJ Sheppard DN Mall MA Galietta LJ Bypassing CFTR dysfunction in cystic fibrosis with alternative pathways for anion transport *Curr Opin Pharmacol* 2017 34 91 97 29065356 10.1016/j.coph.2017.10.002

[30] Shekarabi M Zhang J Khanna AR Ellison DH Delpire E Kahle KT Wnk kinase signaling in ion homeostasis and human disease *Cell Metab* 2017 25 285 299 28178566 10.1016/j.cmet.2017.01.007

[31] Park S Hong JH Ohana E Muallem S The WNK/SPAK and IRBIT/PP1 pathways in epithelial fluid and electrolyte transport *Physiology (Bethesda)* 2012 27 291 299 23026752 10.1152/physiol.00028.2012 PMC3686318

[32] Angyal D Bijvelds MJC Bruno MJ Peppelenbosch MP de Jonge HR Bicarbonate transport in cystic fibrosis and pancreatitis *Cells* 2021 11 54 35011616 10.3390/cells11010054 PMC8750324

[33] Yang D Li Q So I Huang CL Ando H Mizutani A *et al.* IRBIT governs epithelial secretion in mice by antagonizing the WNK/SPAK kinase pathway *J Clin Invest* 2011 121 956 965 21317537 10.1172/JCI43475 PMC3049373

[34] Park HW Nam JH Kim JY Namkung W Yoon JS Lee JS *et al.* Dynamic regulation of CFTR bicarbonate permeability by [Cl-]i and its role in pancreatic bicarbonate secretion *Gastroenterology* 2010 139 620 631 20398666 10.1053/j.gastro.2010.04.004

[35] Kim Y Jun I Shin DH Yoon JG Piao H Jung J *et al.* Regulation of CFTR bicarbonate channel activity by WNK1: implications for pancreatitis and CFTR-related disorders *Cell Mol Gastroenterol Hepatol* 2020 9 79 103 31561038 10.1016/j.jcmgh.2019.09.003 PMC6889609

[36] Dorwart MR Shcheynikov N Wang Y Stippec S Muallem S SLC26A9 is a Cl(-) channel regulated by the WNK kinases *J Physiol* 2007 584 333 345 17673510 10.1113/jphysiol.2007.135855 PMC2277069

[37] Karp PH Moninger TO Weber SP Nesselhauf TS Launspach JL Zabner J *et al.* An in vitro model of differentiated human airway epithelia: methods for establishing primary cultures *Methods Mol Biol* 2002 188 115 137 11987537 10.1385/1-59259-185-X:115

[38] Zabner J Karp P Seiler M Phillips SL Mitchell CJ Saavedra M *et al.* Development of cystic fibrosis and noncystic fibrosis airway cell lines *Am J Physiol Lung Cell Mol Physiol* 2003 284 L844 L854 12676769 10.1152/ajplung.00355.2002

[39] Zabner J Smith JJ Karp PH Widdicombe JH Welsh MJ Loss of CFTR chloride channels alters salt absorption by cystic fibrosis airway epithelia in vitro *Mol Cell* 1998 2 397 403 9774978 10.1016/s1097-2765(00)80284-1

[40] Ramachandran S Krishnamurthy S Jacobi AM Wohlford-Lenane C Behlke MA Davidson BL *et al.* Efficient delivery of RNA interference oligonucleotides to polarized airway epithelia in vitro *Am J Physiol Lung Cell Mol Physiol* 2013 305 L23 L32 23624792 10.1152/ajplung.00426.2012 PMC4073929

[41] Rehman T Karp PH Tan P Goodell BJ Pezzulo AA Thurman AL *et al.* Inflammatory cytokines TNF-α and IL-17 enhance the efficacy of cystic fibrosis transmembrane conductance regulator modulators *J Clin Invest* 2021 131 150398 10.1172/JCI150398 PMC8363270 34166230

[42] McCormick JA Ellison DH The WNKs: atypical protein kinases with pleiotropic actions *Physiol Rev* 2011 91 177 219 21248166 10.1152/physrev.00017.2010 PMC3035565

[43] GTEx Consortium Human genomics. The Genotype-Tissue Expression (GTEx) pilot analysis: multitissue gene regulation in humans *Science* 2015 348 648 660 25954001 10.1126/science.1262110 PMC4547484

[44] Plasschaert LW Žilionis R Choo-Wing R Savova V Knehr J Roma G *et al.* A single-cell atlas of the airway epithelium reveals the CFTR-rich pulmonary ionocyte *Nature* 2018 560 377 381 30069046 10.1038/s41586-018-0394-6 PMC6108322

[45] Montoro DT Haber AL Biton M Vinarsky V Lin B Birket SE *et al.* A revised airway epithelial hierarchy includes CFTR-expressing ionocytes *Nature* 2018 560 319 324 30069044 10.1038/s41586-018-0393-7 PMC6295155

[46] Okuda K Dang H Kobayashi Y Carraro G Nakano S Chen G *et al.* Secretory cells dominate airway cftr expression and function in human airway superficial epithelia *Am J Respir Crit Care Med* 2021 203 1275 1289 33321047 10.1164/rccm.202008-3198OC PMC8456462

[47] Barbry P Marcet B Caballero I Where is the cystic fibrosis transmembrane conductance regulator? *Am J Respir Crit Care Med* 2021 203 1214 1216 33428551 10.1164/rccm.202012-4434ED PMC8456474

[48] Yamada K Park HM Rigel DF DiPetrillo K Whalen EJ Anisowicz A *et al.* Small-molecule WNK inhibition regulates cardiovascular and renal function *Nat Chem Biol* 2016 12 896 898 27595330 10.1038/nchembio.2168

[49] Lu DC Hannemann A Wadud R Rees DC Brewin JN Low PS *et al.* The role of WNK in modulation of KCl cotransport activity in red cells from normal individuals and patients with sickle cell anaemia *Pflugers Arch* 2019 471 1539 1549 31729557 10.1007/s00424-019-02327-7 PMC6892352

[50] Mayes-Hopfinger L Enache A Xie J Huang CL Köchl R Tybulewicz VLJ *et al.* Chloride sensing by WNK1 regulates NLRP3 inflammasome activation and pyroptosis *Nat Commun* 2021 12 4546 34315884 10.1038/s41467-021-24784-4 PMC8316491

[51] Zhang J Deng X Kahle KT Leveraging unique structural characteristics of WNK kinases to achieve therapeutic inhibition *Sci Signal* 2016 9 e3 27811182 10.1126/scisignal.aaj2227

[52] Alessi DR Zhang J Khanna A Hochdörfer T Shang Y Kahle KT The WNK-SPAK/OSR1 pathway: master regulator of cation-chloride cotransporters *Sci Signal* 2014 7 re3 25028718 10.1126/scisignal.2005365

[53] AlAmri MA Kadri H Alderwick LJ Simpkins NS Mehellou Y Rafoxanide and closantel inhibit spak and osr1 kinases by binding to a highly conserved allosteric site on their c-terminal domains *ChemMedChem* 2017 12 639 645 28371477 10.1002/cmdc.201700077

[54] Wine JJ Hansson GC König P Joo NS Ermund A Pieper M Progress in understanding mucus abnormalities in cystic fibrosis airways *J Cyst Fibros* 2018 17 S35 S39 28951068 10.1016/j.jcf.2017.09.003

[55] Smith JJ Travis SM Greenberg EP Welsh MJ Cystic fibrosis airway epithelia fail to kill bacteria because of abnormal airway surface fluid *Cell* 1996 85 229 236 8612275 10.1016/s0092-8674(00)81099-5

[56] Kahle KT Rinehart J Lifton RP Phosphoregulation of the Na-K-2Cl and K-Cl cotransporters by the WNK kinases *Biochim Biophys Acta* 2010 1802 1150 1158 20637866 10.1016/j.bbadis.2010.07.009 PMC3529164

[57] Huang CL Yang SS Lin SH Mechanism of regulation of renal ion transport by WNK kinases *Curr Opin Nephrol Hypertens* 2008 17 519 525 18695394 10.1097/MNH.0b013e32830dd580

[58] Welsh MJ Intracellular chloride activities in canine tracheal epithelium: direct evidence for sodium-coupled intracellular chloride accumulation in a chloride-secreting epithelium *J Clin Invest* 1983 71 1392 1401 6853719 10.1172/JCI110892 PMC437003

[59] Willumsen NJ Davis CW Boucher RC Intracellular Cl- activity and cellular Cl- pathways in cultured human airway epithelium *Am J Physiol* 1989 256 C1033 C1044 2719093 10.1152/ajpcell.1989.256.5.C1033

[60] Willumsen NJ Davis CW Boucher RC Cellular Cl- transport in cultured cystic fibrosis airway epithelium *Am J Physiol* 1989 256 C1045 C1053 2719094 10.1152/ajpcell.1989.256.5.C1045

[61] Sly PD Brennan S Gangell C de Klerk N Murray C Mott L *et al.* Australian Respiratory Early Surveillance Team for Cystic Fibrosis (AREST-CF) Lung disease at diagnosis in infants with cystic fibrosis detected by newborn screening *Am J Respir Crit Care Med* 2009 180 146 152 19372250 10.1164/rccm.200901-0069OC

[62] Schultz A Stick S Early pulmonary inflammation and lung damage in children with cystic fibrosis *Respirology* 2015 20 569 578 25823858 10.1111/resp.12521

[63] Sly PD Gangell CL Chen L Ware RS Ranganathan S Mott LS *et al.* AREST CF Investigators Risk factors for bronchiectasis in children with cystic fibrosis *N Engl J Med* 2013 368 1963 1970 23692169 10.1056/NEJMoa1301725

[64] Lukacs NW Strieter RM Chensue SW Widmer M Kunkel SL TNF-alpha mediates recruitment of neutrophils and eosinophils during airway inflammation *J Immunol* 1995 154 5411 5417 7730642

[65] Karpati F Hjelte FL Wretlind B TNF-alpha and IL-8 in consecutive sputum samples from cystic fibrosis patients during antibiotic treatment *Scand J Infect Dis* 2000 32 75 79 10716082 10.1080/00365540050164263

[66] McAllister F Henry A Kreindler JL Dubin PJ Ulrich L Steele C *et al.* Role of IL-17A, IL-17F, and the IL-17 receptor in regulating growth-related oncogene-alpha and granulocyte colony-stimulating factor in bronchial epithelium: implications for airway inflammation in cystic fibrosis *J Immunol* 2005 175 404 412 15972674 10.4049/jimmunol.175.1.404 PMC2849297

[67] Tan HL Regamey N Brown S Bush A Lloyd CM Davies JC The Th17 pathway in cystic fibrosis lung disease *Am J Respir Crit Care Med* 2011 184 252 258 21474644 10.1164/rccm.201102-0236OC PMC3381840

[68] Rehman T Thornell IM Pezzulo AA Thurman AL Romano Ibarra GS Karp PH *et al.* TNFα and IL-17 alkalinize airway surface liquid through CFTR and pendrin *Am J Physiol Cell Physiol* 2020 319 C331 C344 32432926 10.1152/ajpcell.00112.2020 PMC7500220

[69] Lüscher BP Vachel L Ohana E Muallem S Cl^-^ as a bona fide signaling ion *Am J Physiol Cell Physiol* 2020 318 C125 C136 31693396 10.1152/ajpcell.00354.2019 PMC6985830

[70] Shcheynikov N Son A Hong JH Yamazaki O Ohana E Kurtz I *et al.* Intracellular Cl- as a signaling ion that potently regulates Na+/HCO3- transporters *Proc Natl Acad Sci USA* 2015 112 E329 E337 25561556 10.1073/pnas.1415673112 PMC4311818

[71] Yamaguchi M Steward MC Smallbone K Sohma Y Yamamoto A Ko SB *et al.* Bicarbonate-rich fluid secretion predicted by a computational model of guinea-pig pancreatic duct epithelium *J Physiol* 2017 595 1947 1972 27995646 10.1113/JP273306 PMC5350461

[72] Kim D Huang J Billet A Abu-Arish A Goepp J Matthes E *et al.* Pendrin mediates bicarbonate secretion and enhances cystic fibrosis transmembrane conductance regulator function in airway surface epithelia *Am J Respir Cell Mol Biol* 2019 60 705 716 30742493 10.1165/rcmb.2018-0158OC

[73] Carraro G Langerman J Sabri S Lorenzana Z Purkayastha A Zhang G *et al.* Transcriptional analysis of cystic fibrosis airways at single-cell resolution reveals altered epithelial cell states and composition *Nat Med* 2021 27 806 814 33958799 10.1038/s41591-021-01332-7 PMC9009537

[74] Schultz A Puvvadi R Borisov SM Shaw NC Klimant I Berry LJ *et al.* Airway surface liquid pH is not acidic in children with cystic fibrosis *Nat Commun* 2017 8 1409 29123085 10.1038/s41467-017-00532-5 PMC5680186

[75] Abou Alaiwa MH Beer AM Pezzulo AA Launspach JL Horan RA Stoltz DA *et al.* Neonates with cystic fibrosis have a reduced nasal liquid pH: a small pilot study *J Cyst Fibros* 2014 13 373 377 24418186 10.1016/j.jcf.2013.12.006 PMC4060428

[76] Oh SW Han SY Loop diuretics in clinical practice *Electrolyte Blood Press* 2015 13 17 21 26240596 10.5049/EBP.2015.13.1.17 PMC4520883

[77] Grubb BR Pace AJ Lee E Koller BH Boucher RC Alterations in airway ion transport in NKCC1-deficient mice *Am J Physiol Cell Physiol* 2001 281 C615 C623 11443061 10.1152/ajpcell.2001.281.2.C615

[78] Raju SV Lin VY Liu L McNicholas CM Karki S Sloane PA *et al.* The cystic fibrosis transmembrane conductance regulator potentiator ivacaftor augments mucociliary clearance abrogating cystic fibrosis transmembrane conductance regulator inhibition by cigarette smoke *Am J Respir Cell Mol Biol* 2017 56 99 108 27585394 10.1165/rcmb.2016-0226OC PMC5248967

